# Ulcerative Colitis in a Patient With Auricular Chondritis and Sweet Syndrome: A Rare Case

**DOI:** 10.1155/carm/1251336

**Published:** 2026-08-07

**Authors:** Guang-He Zhang, Hai-Yan Wang, Chun-Fang Guo, Xian-Wei Han

**Affiliations:** ^1^ Department of Dermatology, Shenyang Seventh People’s Hospital, Shenyang, China

**Keywords:** auricular chondritis, relapsing polychondritis, sweet syndrome, ulcerative colitis

## Abstract

Relapsing polychondritis (RP) is a rare immune‐mediated inflammatory disorder primarily affecting cartilaginous tissues throughout the body that may involve other connective tissues. The initial manifestation is often auricular chondritis, and cutaneous presentations are relatively uncommon. Approximately 30% of RP cases have an association with autoimmune diseases, but it is incredibly rare for there to be any coexistence with inflammatory bowel disease (IBD). Sweet syndrome (SS), which is also known as acute febrile neutrophilic dermatosis, also has an association with autoimmune disorders and is considered to be an incredibly rare extraintestinal IBD manifestation. In this article, a case of a patient simultaneously presenting with auricular chondritis, ulcerative colitis (UC), and SS, which had an effective response to systemic corticosteroid therapy is reported. As far as we are aware, this is the first reported case of the coexistence of these three disorders, and it will provide valuable insights into the diagnosis and management of RP that is complicated by UC and other associated conditions.

## 1. Introduction

Relapsing polychondritis (RP) is a rare autoimmune disorder that primarily affects cartilaginous tissues, including the auricles, nose, larynx, and trachea. Other connective tissues can also be involved, such as the eyes, inner ear, heart, blood vessels, and skin [1, 2]. Approximately 30% of RP cases coexist with other autoimmune diseases, including rheumatoid arthritis, systemic lupus erythematosus, and Sjögren’s syndrome, while it is incredibly rare for there to be coexistence with inflammatory bowel disease (IBD) [3, 4]. Sweet syndrome (SS) is characterized by the abrupt onset of tender erythematous nodules or plaques on the skin. This is typically accompanied by fever, arthralgias, and leukocytosis with neutrophilic predominance. A histopathological examination reveals dense neutrophilic infiltrates in the dermis. SS is an inflammatory skin disorder that has a potential association with autoimmune diseases, malignancies, infections, and medications [5]. A case of a patient with ulcerative colitis (UC) that was complicated by auricular chondritis and SS is reported in this article. It is believed that this is the first time that the coexistence of these three conditions has been documented in a case.

## 2. Case Report

A 64‐year‐old male presented with recurrent erythema, swelling, and pain in both of his auricles for 3 years. This was accompanied by left forearm erosion for more than 20 days. The patient had developed bilateral auricular erythema, swelling, and pain without obvious triggers 3 years previously. This did not respond to antibiotics and resolved spontaneously within several days to weeks. 20 days prior to his admission, tender erythema with surrounding pustules developed on his left forearm, which later coalesced into a lesion that was covered with purulent discharge. Three days prior to his admission, two tender erythematous plaques with peripheral pseudovesicles appeared on his posterior neck.

18 months earlier, the patient was diagnosed with UC at a local gastroenterology department due to abdominal pain, diarrhea, and bloody mucopurulent stools. He was maintained on vedolizumab (300 mg every 8 weeks) and in clinical remission. Six months earlier, he underwent surgical resection for a left lung microinvasive adenocarcinoma. No significant allergies or family history of similar diseases were reported. A physical examination revealed there to be no systemic abnormalities. In particular, there were no gastrointestinal symptoms.

A dermatological examination revealed bilateral auricular swelling, induration, tenderness without fluctuance, and sparing of the earlobes. Both auricles were deformed and exhibited a cauliflower ear appearance. An oval superficial erosion with violaceous borders and fused pustules was observed on the left forearm, and it was covered by yellow purulent discharge. At the same time, two coin‐sized, infiltrative erythematous plaques with raised annular edges and miliary pseudovesicles were observed on the posterior neck (Figure 1). Laboratory findings found there to be elevated levels of C‐reactive protein and reduced levels of hemoglobin, while the white blood cell and neutrophil counts were both normal. ANA was positive at 1:320 (normal: negative), and anti‐Scl‐70 antibody was positive (++). The results of stool tests were also normal. The chest CT showed postoperative changes in the left lung without airway abnormalities. The colonoscopy was deferred due to the absence of abdominal symptoms. The patient declined auricular biopsy. Histopathology of the left forearm lesion showed there to be diffuse neutrophilic infiltration of the upper reticular dermis (Figure 2). Periodic acid–Schiff (PAS) and acid‐fast staining were both negative.

**FIGURE 1:**
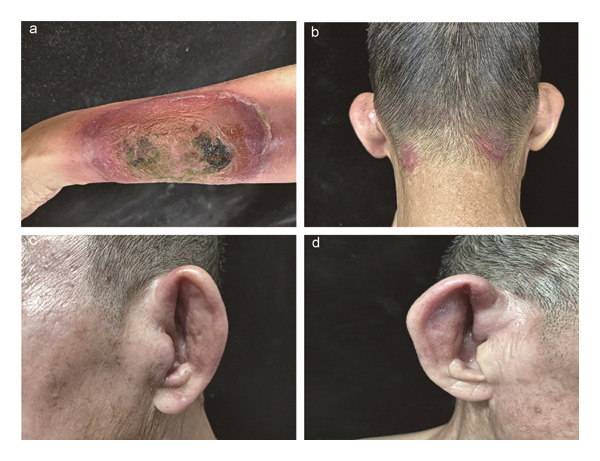
Skin lesions. Clinical findings included erythematous plaques on the left forearm (a) and posterior neck (b), and erythema of the cartilaginous portion of both pinnae [(c) right and (d) left], with sparing of the earlobes.

**FIGURE 2:**
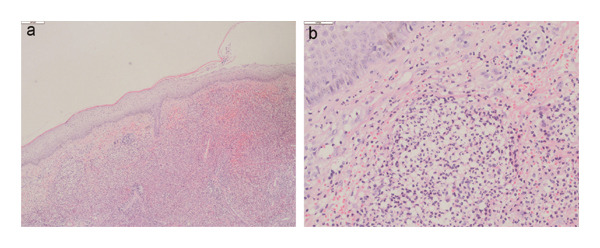
Histopathological findings. Subcorneal pustules are observed, containing neutrophils with transepidermal migration. Dermal vessels exhibit dilation without fibrinoid necrosis or neutrophilic infiltration in their walls. A diffuse neutrophilic infiltrate involves the superficial to mid dermis, accompanied by prominent nuclear dust and extensive erythrocyte extravasation. (a) (HE × 40); (b) (HE × 200).

The patient was diagnosed with auricular chondritis and SS. His treatment included 30 mg/day of systemic corticosteroids (equivalent to prednisone 0.500 mg/kg/day), daily wound care, and wet compresses with benzalkonium bromide. After 1 week, the forearm ulcer had decreased in size with crust formation, the neck plaque had resolved, and the auricular swelling had improved significantly. After 2 weeks of systemic steroid therapy, the skin lesions of the patient had completely resolved, in addition to the resolution of erythema and ear swelling. The steroid dose was reduced to 22.5 mg/day (equivalent to prednisone 0.375 mg/kg/day). One month after the patient was discharged, the dose was tapered to 5 mg/day (equivalent to prednisone 0.083 mg/kg/day), and this was maintained for 1 week before being discontinued. Telephone follow‐up at 3 months postdischarge revealed there to be no recurrence of skin lesions or auricular swelling (Figure 3). The patient remains under follow‐up.

**FIGURE 3:**
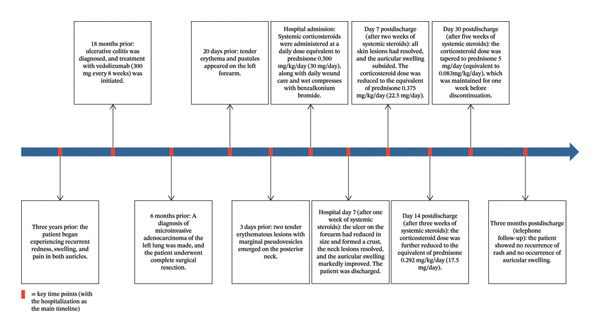
Concise timeline. A timeline was constructed with the hospitalization period of the patient as the primary time node, spanning from the onset of bilateral auricular symptoms 3 years before admission to the 3‐month follow‐up after discharge, and anchored by the emergence of key clinical symptoms and major therapeutic interventions.

## 3. Discussion

Auricular chondritis is the most common manifestation of RP, and it occurs in approximately 90% of patients, often as the initial symptom [2]. Acute episodes present with recurrent erythema, swelling, and pain in the auricles (sparing the earlobes). Chronic inflammation can lead to cartilage destruction, fibrosis, and permanent deformities. This is often referred to as “cauliflower ear” [6]. Progressive disease may cause the transient or permanent impairment of hearing [6].

RP is incredibly rare and has an estimated prevalence of a very few per million [7]. There are no specific laboratory or histopathological markers for RP, and its diagnosis is reliant on clinical criteria [4]. The original diagnostic criteria that were established by McAdam et al. [8] suggested that a patient was highly likely to have RP if exhibiting three or more of the six diagnostic features, which included auricular chondritis, nasal chondritis, laryngotracheal chondritis, seronegative inflammatory arthritis, ocular inflammation, and cochlear/vestibular dysfunction. Damiani and Levine [9] later revised the diagnostic criteria and incorporated the histopathologic confirmation of lesional cartilage or a positive response to glucocorticoids or dapsone therapy as additional diagnostic components. Auricular chondritis was the initial manifestation in this patient. This condition can easily be misdiagnosed as auricular perichondritis, which is typically an acute suppurative inflammation of the auricular perichondrium that is caused by trauma or infection, and often presents unilaterally and responds well to antibiotic therapy. However, this patient had no history of trauma or infection involving the ear, and multiple courses of antibiotic treatment proved to be ineffective, which made auricular perichondritis unlikely. Although this patient did not present with nasal chondritis, laryngotracheal chondritis, seronegative inflammatory arthritis, ocular inflammation, or cochlear/vestibular dysfunction—meaning that there was a lack of sufficient evidence for an RP diagnosis—they exhibited long‐term recurrent erythema, swelling, and pain of both auricles, with typical cauliflower ear deformity, and responded well to glucocorticoid therapy. A diagnosis of auricular chondritis secondary to RP was considered based on the Damiani diagnostic criteria. The lack of a cartilage biopsy from the ear is a limitation of this case. Long‐term monitoring is recommended for other potential RP manifestations. RP and IBD are both immune‐mediated diseases. RP has been reported to be an incredibly rare extraintestinal manifestation (EIM) of IBD [10]. Over 30 cases of coexisting RP and IBD have been documented to date [4, 6, 11].

RP treatment is empirical and tailored to disease activity and organ involvement [4]. There may be a response to NSAIDs, dapsone, or low‐dose corticosteroids in mild cases, while high‐dose corticosteroids or immunosuppressants (e.g., methotrexate, cyclosporine, and azathioprine) are required in severe cases, such as those with airway involvement [11]. TNF‐α inhibitors have proven to be effective for refractory cases [4, 11, 12].

SS is characterized by the sudden onset of tender erythematous plaques, which are typically found on the face, neck, or arms, sometimes with pseudovesicles, ulcers, or pustules. A histopathological examination reveals dense neutrophilic infiltrates in the dermis, usually without any evidence of primary leukocytoclastic vasculitis. The skin lesions of this patient presented as painful erythema with sudden onset on the left forearm and neck, and pseudovesicles were observed around the erythema on the neck. Pathological biopsy of the margin of the left forearm ulcer found subcorneal pustules that contained neutrophilic transepidermal migration, dilation of dermal blood vessels without fibrinoid necrosis or neutrophilic infiltration of the vessel walls, and diffused neutrophilic infiltration that involved the superficial to mid‐dermis, together with prominent nuclear dust and extensive erythrocyte extravasation. These findings are consistent with the cutaneous and pathological manifestations of SS. In addition, the patient had comorbid malignancies and IBD, and the skin rash significantly improved after systemic corticosteroid therapy. These features fulfill the SS diagnostic criteria [13]. SS must be differentiated from pyoderma gangrenosum (PG), as they are both neutrophilic diseases that can present as EIMs of IBD or cutaneous manifestations in RP patients. The lower extremities and peristomal areas are commonly involved in the latter condition, and painful penetrating ulcers that may extend deep into the dermis or subcutaneous tissue that often heal with scar formation typically present in this scenario. The skin lesions of this patient were confined to the upper limbs and neck, and there was no evidence of deep ulceration. Furthermore, the cervical plaque was completely resolved, and the superficial ulcer on the left forearm healed rapidly with crust formation and no subsequent scarring following systemic corticosteroid therapy. It is important to note that SS also requires differentiation from several cutaneous infectious and inflammatory diseases [14], such as erysipelas, cellulitis, leprosy, fungal infections, and panniculitis, as these conditions can share some common clinical features, which include fever, leukocytosis, painful erythema, and pustules. There was no fever or elevated white blood cell count in this patient. The histopathological findings were consistent with SS, and PAS staining and acid‐fast staining were negative. The patient also showed good responsiveness to glucocorticoid therapy. Therefore, we are more in favor of the SS diagnosis.

Approximately 20% of SS cases are considered to have an association with malignant tumors, primarily hematologic malignancies, while others are solid tumors, including gastrointestinal tumors, urologic tumors, and breast cancer [15]. SS is incredibly rare in lung cancer, and little more than 20 cases of lung cancer‐associated SS have been reported to date. It is notable that the present patient had undergone the complete resection of the pulmonary tumor 6 months before SS onset. Furthermore, repeat chest CT showed no evidence of tumor recurrence at the time of SS diagnosis. Cutaneous manifestations occur in 35%–50% of RP patients, while SS is relatively rare, with just over 20 reported cases of concurrent SS and RP to date [1, 2, 16]. Some scholars have suggested that the incidence of complications is significantly higher when SS and RP coexist in the same patient, which warrants vigilance regarding hematologic malignancy development [1, 2, 16]. SS occurs in 0.07%–0.21% of IBD patients [17]. UC activity assessment should be based on objective indicators [18, 19]. This patient had no current symptoms of abdominal pain, diarrhea, or bloody mucoid stools, and the results of a routine stool examination and fecal occult blood test were normal. Although the C‐reactive protein level was elevated and the hemoglobin level was decreased, these findings lacked specificity, and therefore, active UC could not be confirmed. The absence of endoscopic examination and fecal calprotectin testing is a limitation of this study. The patient is currently receiving 300 mg of vedolizumab every 8 weeks. Although most SS activity parallels that of IBD, only a subset of patients on biologic therapy had active IBD when diagnosed with SS [17]. Therefore, the SS may represent a cutaneous manifestation that is attributable to the combined effects of RP and/or IBD in this case. The first‐line therapeutic agents for SS typically include glucocorticoids, colchicine, and potassium iodide, while second‐line treatments include dapsone, cyclosporine, clofazimine, and indomethacin. Various biologics and small‐molecule drugs have often been used in recent years for the management of refractory, recurrent cases or those with inadequate response to systemic corticosteroid therapy. Biologics represent an important and effective therapeutic option, particularly in patients with concomitant IBD [17, 20].

In summary, a case of UC maintained in clinical remission with vedolizumab, where the patient was subsequently diagnosed with auricular chondritis and SS has been reported in this study. It remains unclear whether these represent EIMs of UC or independent comorbid conditions, although the cutaneous lesions responded well to systemic corticosteroid therapy. When auricular chondritis and SS occur concomitantly in this patient, vigilance should be maintained for the development of malignancies, particularly hematologic malignancies, and they should be monitored closely for other potential manifestations of RP.

## 4. Conclusion

RP can involve multiple cartilaginous systems, which can lead to varied initial presentations across different specialties. Misdiagnosis is common due to its rarity. Auricular chondritis is a frequent initial manifestation of RP that necessitates heightened awareness among dermatologists and otolaryngologists and close monitoring for subsequent systemic involvement. RP is currently considered to be an EIM of IBD. When clinicians encounter IBD patients who present with recurrent redness and swelling of the auricular cartilage or recurrent inflammation that involves cartilage at other sites, the coexisting RP possibility should be considered. Increased awareness of this condition will facilitate early diagnosis and treatment, in addition to enabling the monitoring and prevention of more serious complications. SS can occur as an independent disease or as a cutaneous manifestation of RP and/or IBD. All three of these conditions are immune‐mediated disorders, and future research should examine whether their pathogeneses are correlated.

## Funding

No funds were received.

## Consent

Written signed informed consent was obtained from the patient prior to writing the manuscript.

## Conflicts of Interest

The authors declare no conflicts of interest.

## Data Availability

The data that support the findings of this study are available from the corresponding author upon reasonable request.
